# Effect of Sitting Position vs. Supine Position With the Head Turned to the Affected Side on Benign Paroxysmal Positional Vertigo Fatigue

**DOI:** 10.3389/fneur.2021.705034

**Published:** 2021-06-17

**Authors:** Takao Imai, Suetaka Nishiike, Tomoko Okumura, Noriaki Takeda, Takashi Sato, Yumi Ohta, Takefumi Kamakura, Hidenori Inohara

**Affiliations:** ^1^Department of Otorhinolaryngology – Head and Neck Surgery, Osaka University Graduate School of Medicine, Suita, Japan; ^2^Department of Otorhinolaryngology – Head and Neck Surgery, Osaka Rosai Hospital, Sakai, Japan; ^3^Department of Otorhinolaryngology – Head and Neck Surgery, Tokushima University Graduate School of Medicine, Tokushima, Japan

**Keywords:** canalolithiasis, Dix-Hallpike test, positional nystagmus, sitting position, BPPV fatigue

## Abstract

**Objective:** In benign paroxysmal positional vertigo (BPPV), positional nystagmus becomes generally weaker when the Dix–Hallpike test is repeated. This phenomenon is termed BPPV fatigue. We previously reported that the effect of BPPV fatigue deteriorates over time (i.e., the positional nystagmus is observed again after maintaining a sitting head position). The aim of this study was to investigate whether the effect of BPPV fatigue attenuates after maintaining a supine position with the head turned to the affected side.

**Methods:** Twenty patients with posterior-canal-type BPPV were assigned to two groups. Group A received Dix–Hallpike test, were returned to the sitting position (reverse Dix–Hallpike test) with a sitting head position for 10 min, and then received a second Dix–Hallpike test. Group B received Dix–Hallpike test, were kept in the supine position with the head turned to the affected side for 10 min, and then received reverse Dix–Hallpike test followed by the second Dix–Hallpike test. The maximum slow phase eye velocity (MSPEV) of positional nystagmus induced by the first, reverse, and second Dix–Hallpike test were analyzed.

**Results:** The ratio of MSPEV of the positional nystagmus induced by the second Dix–Hallpike test relative to the first Dix–Hallpike test was significantly smaller in group B than that in group A. There was no difference in the MSPEV of the positional nystagmus induced by the reverse Dix–Hallpike test between group A and B.

**Conclusions:** The effect of BPPV fatigue is continued by maintaining a supine position with the head turned to the affected side, while the effect is weakened by maintaining a sitting head position. On the basis of the most widely accepted theory of the pathophysiology of BPPV fatigue, in which the particles become dispersed along the canal during head movement in the Dix–Hallpike test, we found an inconsistency whereby the dispersed otoconial debris return to a mass during the sitting position but do not return to a mass in the supine position with the head turned to the affected side. Future studies are required to determine the exact pathophysiology of BPPV fatigue.

**Classification of Evidence:** 2b.

## Introduction

Patients with posterior-canal-type benign paroxysmal positional vertigo (BPPV) exhibit characteristic positional nystagmus induced by the Dix–Hallpike test, in which the patient is brought from the upright to the supine head-hanging position with the head turned 45° to the affected ear (1). However, the vertigo and positional nystagmus are generally weaker when the Dix–Hallpike test is repeated, a phenomenon termed BPPV fatigue (2), which is a specific characteristic of BPPV. We previously reported that the effect of BPPV fatigue is reduced when the head is maintained in the sitting position; i.e., the intensity of positional nystagmus induced by the Dix–Hallpike test was reduced by BPPV fatigue, and the intensity of nystagmus recovered over time (3). When performing the Dix–Hallpike test, patients are placed in either the sitting upright position or the head-hanging position with the head turned 45° to the affected ear. The aim of the present study was to examine whether the effect of BPPV fatigue is reduced by maintaining the head in the supine position with the head turned to the affected side and examine the pathophysiology of BPPV fatigue.

## Materials and Methods

All procedures involving human participants were performed in accordance with the ethical standards of the institutional and/or national research committee and the 1964 Helsinki Declaration and its later amendments or comparable ethical standards. Approval for the study was obtained from the ethics committees of Osaka Rosai Hospital and Osaka University Hospital (No. 16165), and the study was included in the University Hospital Medical Information Network (UMIN) (UMIN000025291). Before experiments, written informed consent was obtained from all participants included in the study.

**Figure 1:**
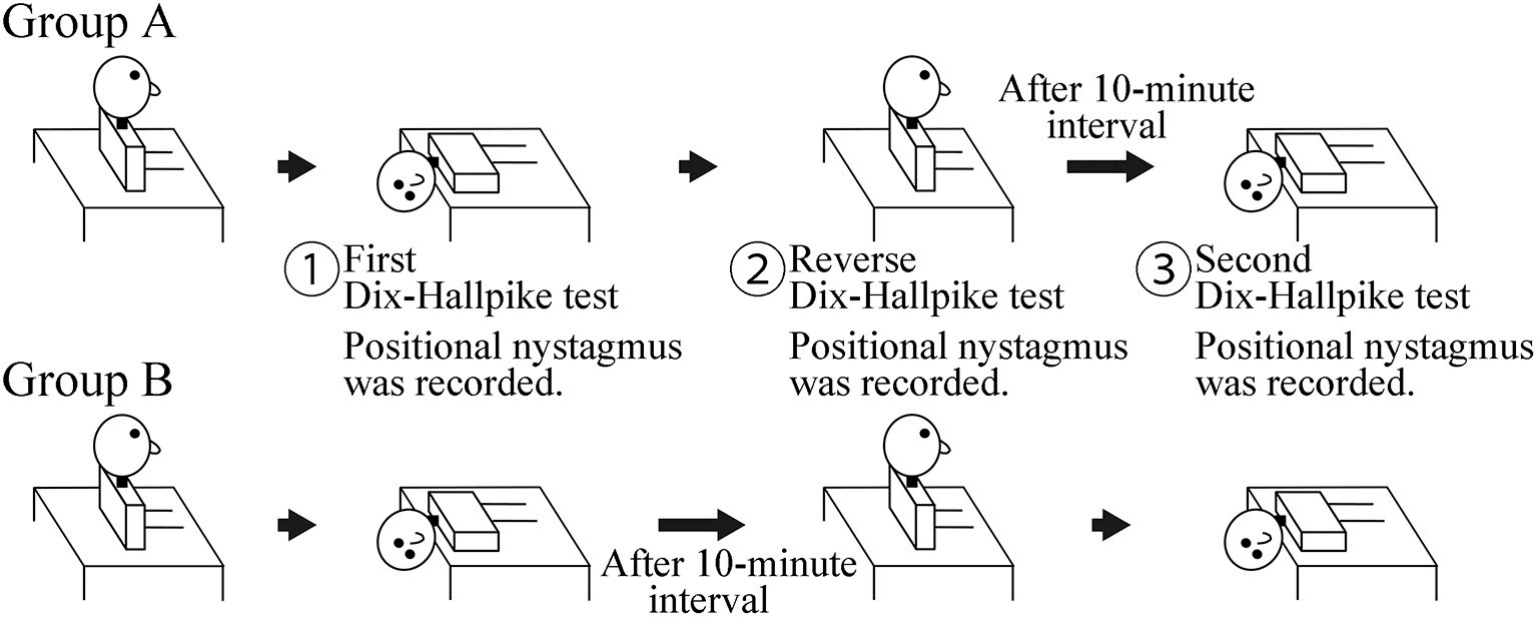
Schematic of the study. Group A: a 10-min interval was set in the sitting position after the reverse Dix–Hallpike test. Group B: a 10-min interval was set in the supine position with the head turned to the affected side after the first Dix–Hallpike test. Positional nystagmus was recorded at the head positions shown by ①, ②, and ③.

This was a prospective observational study. The study included 20 patients (seven men and 13 women; 48–88 years old; median age, 69 years; right ear affected in 11, left ear affected in nine) with canalolithiasis of the posterior canal type of BPPV (1) at the Department of Otorhinolaryngology—Head and Neck Surgery, Osaka Rosai Hospital or Osaka University Hospital between January 2014 and December 2016. All patients underwent two consecutive Dix–Hallpike tests on the affected side ear (4). Ten patients at Osaka University Hospital were allocated to group A, and 10 patients at Osaka Rosai Hospital were allocated to group B. Group A had a 10-min interval time in the sitting position after a reverse Dix–Hallpike test (4). Group B had a 10-min interval time in the supine position with the head turned to the affected side. The details are shown in Figure 1.

For the Dix–Hallpike tests, patients wore goggles with an infrared charge-coupled device camera (RealEyes; Micromedical Technologies, Chatham, IL, USA). The positional nystagmus in the supine position with the head turned to the affected side at the first (① in Figure 1) and second (③ in Figure 1) Dix–Hallpike test and that in the sitting position after the reverse Dix–Hallpike test (② in Figure 1) were recorded on a Windows computer with USB-connected video capture (GV-USB2; I-O DATA, Ishikawa, Japan). One author (TI) performed the two Dix–Hallpike tests in all patients. The doctor was certified to have expert knowledge and medical technique to treat patients with a complaint of dizziness and/or vertigo; this certification was provided by the Japan Society for Equilibrium Research. Patients were instructed to keep their eyes open and continue looking forward in the dark during the test. After data collection, patients received the Epley maneuver (4, 5).

One author (TO) who was blinded to the patients' data analyzed the eye movement three-dimensionally (3D) to determine the maximum slow-phase eye velocity (MSPEV) of the positional nystagmus. The ratio of the MSPEV of positional nystagmus induced by the second Dix–Hallpike test against that induced by first Dix–Hallpike test in both groups A and B was calculated, as was the ratio of the MSPEV of positional nystagmus induced by the reverse Dix–Hallpike test against that induced by the first Dix–Hallpike test in both groups A and B.

### 3D Analysis of Eye Movements

**Figure 2:**
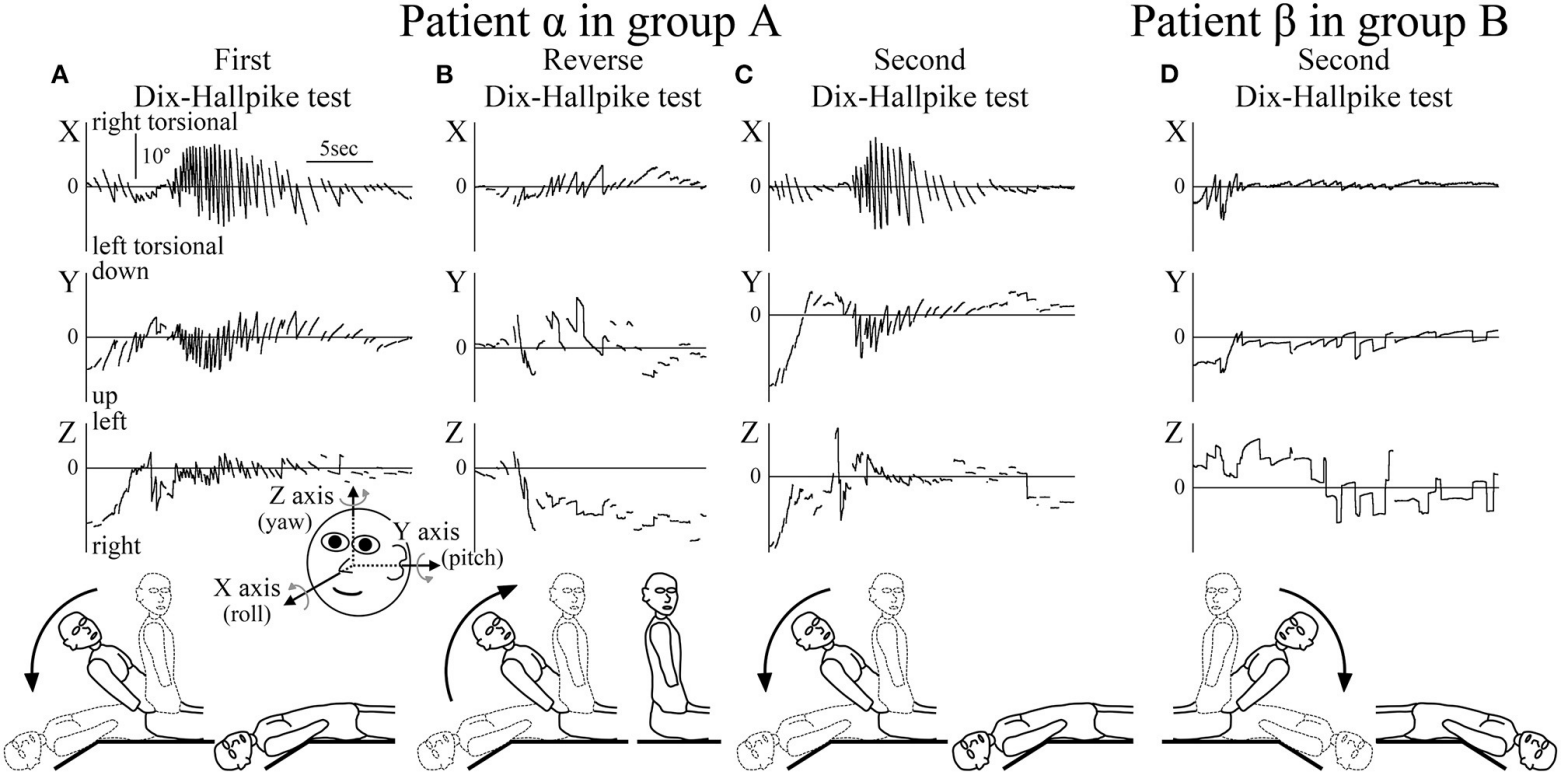
Axis angles of the eye position of the positional nystagmus. **(A–C)** Patient α allocated to group A. **(D)** Patient β allocated to group B. **(A)** Axis angle of the eye position of the positional nystagmus induced by the first right Dix–Hallpike test. A strong right torsional (X component), upward (Y component), and leftward (Z component) positional nystagmus was observed. Inserted figure of the face shows the three-dimensional (3D) coordinates used. **(B)** Axis angle of the eye position of the positional nystagmus induced by the reverse Dix–Hallpike test. Left torsional and downward positional nystagmus were observed. The direction of the positional nystagmus was opposite to that induced by the first right Dix–Hallpike test **(A)**. **(C)** Axis angle of the eye position of the positional nystagmus induced by the second right Dix–Hallpike test. A similar strong positional nystagmus to that induced by the first Dix–Hallpike test **(A)** was observed. **(D)** Axis angle of the eye position of the positional nystagmus induced by the second left Dix–Hallpike test. In patient β (allocated to group B), the positional nystagmus induced by the second Dix–Hallpike test was much weaker than that in patient α (group A; Figure 2C). The nystagmus observed at the initial part was not positional but rather nystagmus induced by the vestibulo-ocular reflex (VOR) during head movement of the second left Dix–Hallpike test.

The 30-Hz eye movement images were converted to 720 × 480-pixel JPEG images and analyzed with an algorithm developed in our laboratory (6, 7). The head coordinates were reconstructed in 3D and defined as follows: the X-axis was parallel to the naso-occipital axis (positive forward), the Y-axis was parallel to the interaural axis (positive left), and the Z-axis was normal to the X–Y plane (positive upwards) (inserted figure in Figure 2A). The axis angle of the eye position was calculated (8, 9). The X, Y, and Z components of the axis angle of the eye position primarily reflected the roll, pitch, and yaw components, respectively (inserted figure in Figure 2A). The direction of rotation was described from the participants' point of view. The accuracy of this method of analyzing eye axis angles was previously described (6, 10). We calculated the axis angle of eye velocity around the X-, Y-, and Z-axes (11). We then extracted the slow-phase eye velocity (SPEV) of the nystagmus using a fuzzy set-based approach (12, 13) and determined the MSPEV of the positional nystagmus (3, 14).

### Statistical Analysis

**Figure 3:**
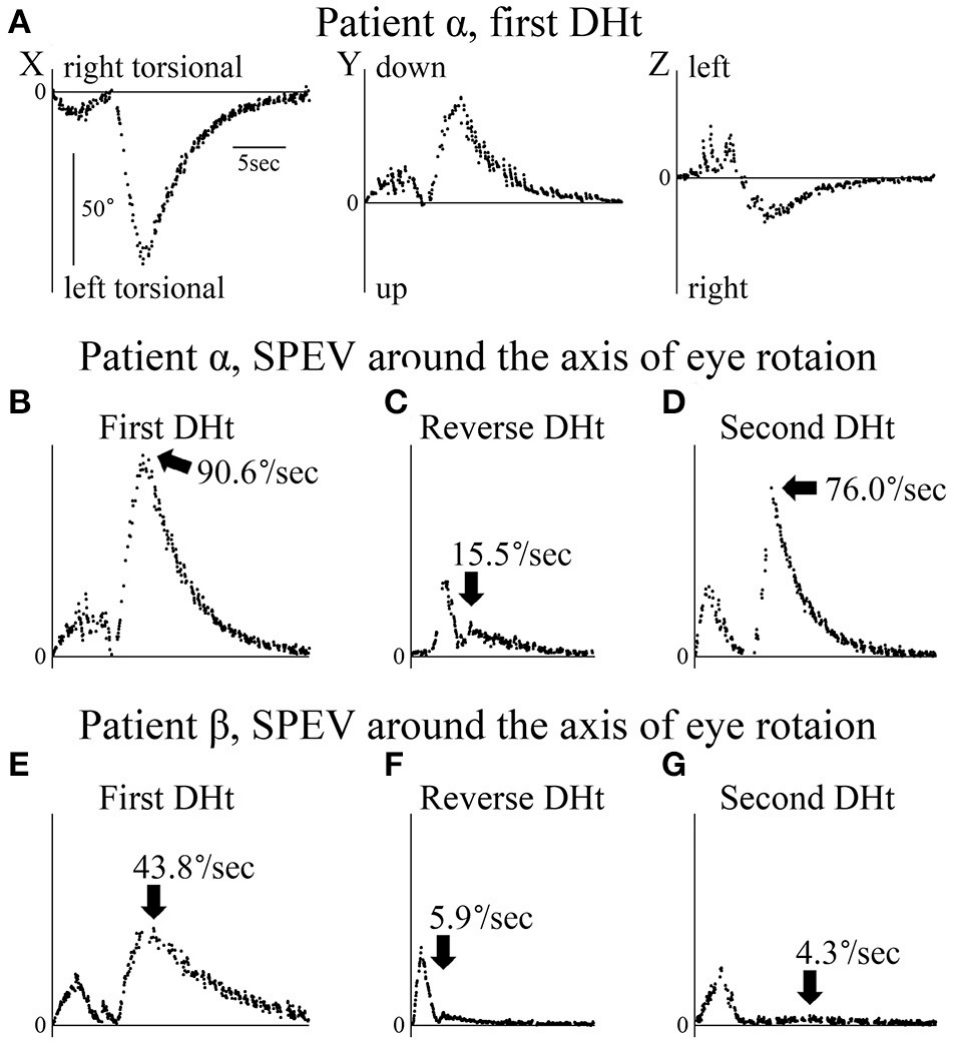
Axis angles of the slow phase eye velocity (SPEV) of the positional nystagmus. DHt, Dix–Hallpike test. **(A–D)** Patient α allocated to group A. (**E–G**) Patient β allocated to group B. (**A**) 3D axis angle of the SPEV of the positional nystagmus induced by the first right Dix–Hallpike test. These data were calculated from the eye position data shown in Figure 2A. In the X and Y components, two peaks were observed. The first peak was caused by the VOR, while the second was caused by the positional nystagmus. After the second peak, the SPEVs in X, Y, and Z components were reduced; the SPEV became almost 0 after 16 s. Thus, the positional nystagmus disappeared at 16 s after the peak SPEV. (**B**) Axis angle of the SPEV of the positional nystagmus around the axis of the eye rotation induced by first right Dix–Hallpike test. This is the absolute value of the 3D vector of the axis angle of the SPEV of the positional nystagmus shown in **Panel A**. This value reflects the magnitude of the SPEV around the axis of eye rotation. Two peaks were observed. The first peak was caused by the VOR, while the second peak was caused by the positional nystagmus. The maximum SPEV (MSPEV) of the positional nystagmus was 90.6°/s. (**C**) Axis angle of the SPEV of the positional nystagmus around the axis of eye rotation induced by the reverse Dix–Hallpike test. These data were calculated from the eye position data shown in Figure 2B. The MSPEV of the positional nystagmus was 15.5°/s. **(D)** Axis angle of the SPEV of the positional nystagmus around the axis of eye rotation induced by the second right Dix–Hallpike test. These data were calculated from the eye position data shown in Figure 2C. The MSPEV of the positional nystagmus was 76.0°/s. **(E)** Axis angle of the SPEV of the positional nystagmus around the axis of eye rotation induced by the first left Dix–Hallpike test. The MSPEV of the positional nystagmus was 43.8°/s. **(F)** Axis angle of SPEV of the positional nystagmus around the axis of eye rotation induced by the reverse Dix–Hallpike test. The MSPEV of the positional nystagmus was 5.9°/s. **(G)** Axis angle of the SPEV of the positional nystagmus around the axis of eye rotation induced by the second left Dix–Hallpike test. These data were calculated from the eye position data shown in Figure 2D. The MSPEV of the positional nystagmus was 4.3°/s.

Statistical analysis was performed using statistical software (BellCurve for Excel; Social Survey Research Information Co., Ltd., Tokyo, Japan). In both groups A and B, differences in the MSPEVs of the positional nystagmus were compared for statistical significance using the Friedman test, a non-parametric test for testing differences between several related samples; the Wilcoxon signed-rank test was used for *post-hoc* analysis. Differences in the ratio of the MSPEV of the positional nystagmus induced by the second Dix–Hallpike (Figure 1, ③) test against that induced by the first Dix–Hallpike test (Figure 1, ①) between group A and B were compared using the Mann–Whitney *U*-test. This ratio is an indicator of the effect of BPPV fatigue; a small ratio indicates a strong effect, while a large ratio indicates a weak effect. Differences in the ratio of the MSPEV of the positional nystagmus induced by the reverse Dix–Hallpike test (Figure 1, ②) against that induced by the first Dix–Hallpike test (Figure 1, ①) between group A and B were compared using the Mann–Whitney *U*-test. Statistical significance was defined as *p* < 0.05.

## Results

The 3D axis angles of the eye position of the positional nystagmus induced by the first Dix–Hallpike test, reverse Dix–Hallpike test, and second Dix–Hallpike test in a representative patient α (male, 80 s, right side affected) allocated to group A are shown in Figures 2A–C. Weak nystagmus was observed (initial part of Figure 2A) during head movement in the first right Dix–Hallpike test (Figure 1, ①), which resulted from the vestibulo-ocular reflex (VOR) induced by head movement. After the head reached the right head-hanging position, a strong right torsional (X component), upward (Y component), and leftward (Z component) nystagmus was observed. Weak nystagmus resulting from VOR (initial part of Figure 2B) was observed during head movement in the reverse Dix–Hallpike test (Figure 1, ②). After the head reached the sitting position, a weak left torsional and downward nystagmus was observed. The direction of the nystagmus was opposite to that induced by the first Dix–Hallpike test (Figure 2A). A weak nystagmus resulting from VOR (initial part of Figure 2C) was observed during head movement in the second right Dix–Hallpike test (Figure 1, ③). After the head reached the right head-hanging position, a similar strong right torsional, upward, and leftward nystagmus to that induced by the first Dix–Hallpike test (Figure 2A) was observed. By contrast, in patient β (male, 40 s, left side affected; group B), the intensity of positional nystagmus induced by the second left Dix–Hallpike test was very weak, although, nystagmus resulting from VOR was observed (Figure 2D).

The axis angle of the SPEV of the positional nystagmus induced by the first right Dix–Hallpike test in patient α is shown in Figure 3. Two peaks were observed in the X and Y components. The first peak was caused by VOR, while the second was caused by positional nystagmus. After the second peak, SPEVs in the X, Y, and Z components were reduced; the SPEV became almost 0 after 16 s. Thus, 16 s after the peak SPEV, the positional nystagmus disappeared. These results indicate that after reaching the affected side head-hanging position, the right torsional and upward nystagmus appeared with a short latency. Furthermore, the intensity of the nystagmus increased and then decreased, lasting for 16 s. These are typical characteristics observed in the positional nystagmus of patients with right-side affected BPPV (1, 4, 15).

The value of the axis angle of the SPEV (i.e., SPEV around the eye rotational axis) of the positional nystagmus of patient α at the first right Dix–Hallpike test is shown in Figure 3B. The value was calculated using the following formula:

$$\begin{array}{l} \sqrt{{\left(X\;component\;of\;axis\;angle\;of\;SPEV\right)}^{2}+{\left(Y\;component\;of\;axis\;angle\;of\;SPEV\right)}^{2}+{\left(Z\;component\;of\;axis\;angle\;of\;SPEV\right)}^{2}} \end{array}$$

Two peaks were seen. The first peak resulted from VOR, while the second peak resulted from positional nystagmus, and the peak SPEV of the positional nystagmus (90.6°/s) was observed. The second peak value was designated as the MSPEV of the positional nystagmus. The axis angle of the SPEV of the positional nystagmus induced by the reverse Dix–Hallpike test in patient α is shown in Figure 3C. The MSPEV of the positional nystagmus was 15.5°/s. The ratio of the MSPEV of the positional nystagmus induced by the reverse Dix–Hallpike test against that induced by the first Dix–Hallpike test was 0.17 (15.5/90.6). The axis angle of the SPEV of the positional nystagmus induced by the second right Dix–Hallpike test in patient α is shown in Figure 3D. The MSPEV of the positional nystagmus was 76.0°/s. The ratio of the MSPEV of the positional nystagmus induced by the second Dix–Hallpike test against that induced by the first Dix–Hallpike test was 0.84 (76.0/90.6).

The axis angles of the SPEV of the positional nystagmus for patient β are shown in Figures 3E–G. The ratio of the MSPEV of the positional nystagmus induced by the reverse Dix–Hallpike test (5.9°/s; Figure 3F) against that induced by the first Dix–Hallpike test (43.8°/s; Figure 3E) was 0.13 (5.9/43.8), close to that of patient α (0.17). The ratio of the MSPEV of the positional nystagmus induced by the second Dix–Hallpike test (4.3°/s; Figure 3G) against that induced by the first Dix–Hallpike test was 0.10 (4.3/43.8), which was much smaller than that for patient α (0.84).

**Figure 4:**
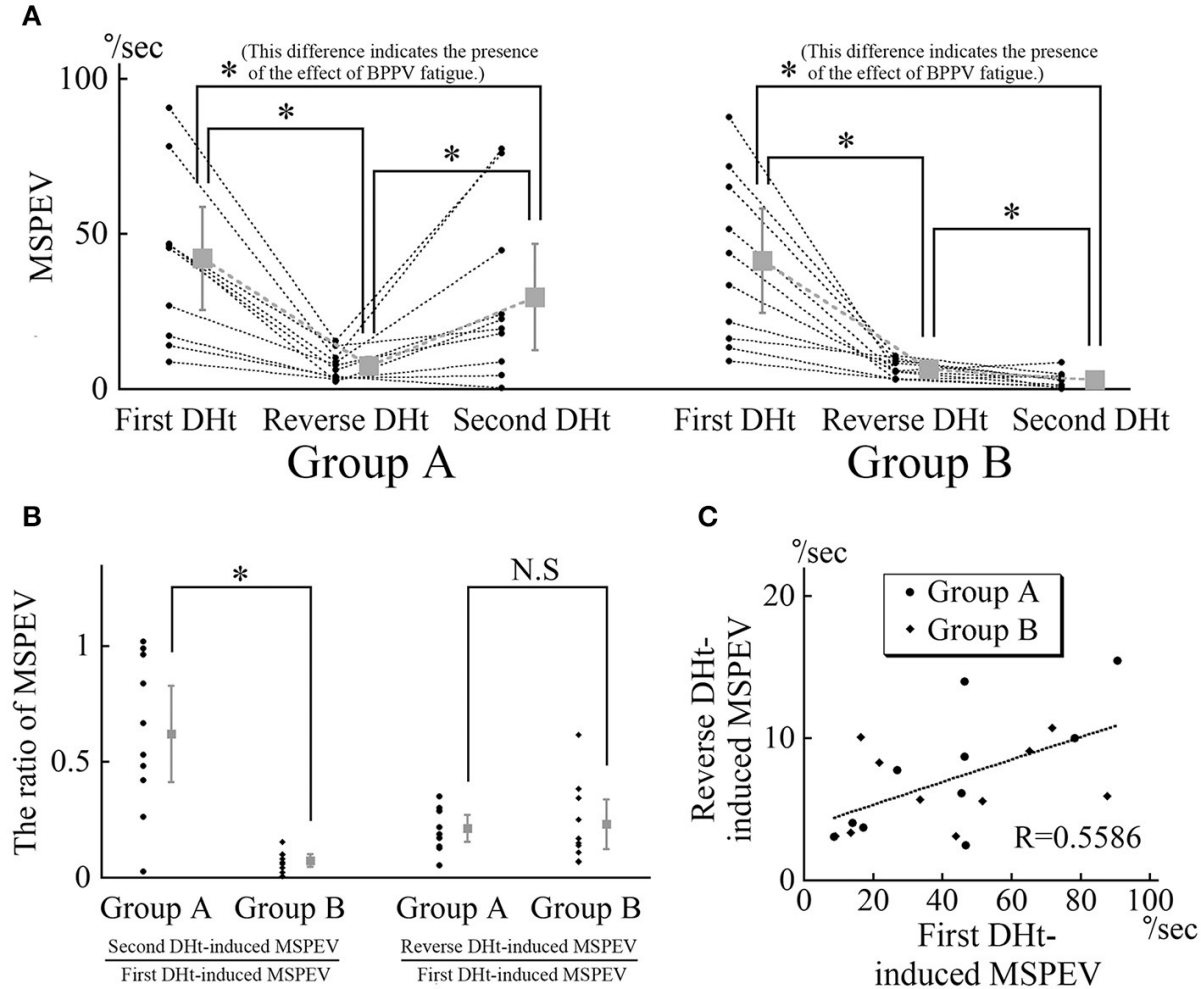
MSPEVs of the positional nystagmus in 10 patients allocated to group A and 10 patients allocated to group B. **(A)** MSPEVs of the positional nystagmus induced by the first, reverse, and second Dix–Hallpike tests in groups A and B. In both groups A and B, there were significant differences in all combinations of MSPEVs. The difference in the MSPEV of the positional nystagmus between that induced by the first and second Dix–Hallpike tests indicates an effect of benign paroxysmal positional vertigo (BPPV) fatigue. **(B)** The ratio of the MSPEV of the positional nystagmus against the MSPEV of the positional nystagmus induced by the first Dix–Hallpike test. Left graph: the ratio of the MSPEV of the positional nystagmus induced by the second Dix–Hallpike test against that induced by first Dix–Hallpike test. The ratio in group B was significantly smaller than that in group A. Right graph: the ratio of the MSPEV of the positional nystagmus induced by the reverse Dix–Hallpike test against that induced by the first Dix–Hallpike test. There were no differences in the ratio between in groups A and B. **(C)** The relationship between the magnitude of the MSPEV of the positional nystagmus induced by the first Dix–Hallpike test and that induced by the reverse Dix–Hallpike test. The graph was obtained by plotting the MSPEV of the positional nystagmus induced by the first Dix–Hallpike test on the horizontal axis and that induced by the reverse Dix–Hallpike test on the vertical axis. The MSPEV of the positional nystagmus induced by the reverse Dix–Hallpike test increased proportionally to the increase induced by the first Dix–Hallpike test in both groups A and B.

The MSPEVs of the positional nystagmus for all 20 patients are shown in Figure 4A. In group A, the average MSPEVs induced by the first, reverse, and second Dix–Hallpike tests were 42.1, 7.5, and 29.6°/s, respectively. Friedman's test showed a significant difference (*p* < 0.0001). Maximum SPEVs between the first and second Dix–Hallpike tests were significantly different (*p* = 0.0069, Wilcoxon signed-rank test). These data indicate that the intensity of the positional nystagmus induced by the second Dix–Hallpike test was weaker than that induced by the first Dix–Hallpike test; i.e., the effect of BPPV fatigue continued. The maximum SPEVs between the first and reverse Dix–Hallpike tests (*p* = 0.0051) and between the second and reverse Dix–Hallpike tests (*p* = 0.0093) were also significantly different. In group B, the average maximum SPEVs during the first, reverse, and second Dix–Hallpike tests were 41.4, 6.5, and 3.0°/s, respectively. Friedman's test showed a significant difference (*p* < 0.0001). The maximum SPEVs between the first and second Dix–Hallpike tests were significantly different (*p* = 0.0051, Wilcoxon signed-rank test). These data indicate that that the intensity of the positional nystagmus induced by the second Dix–Hallpike test became weaker than that induced by the first Dix–Hallpike test; i.e., the effect of BPPV fatigue continued. The maximum SPEVs between the first and reverse Dix–Hallpike tests (*p* = 0.0051) and between the second and reverse Dix–Hallpike tests (*p* = 0.0284) were also significantly different. The effect of BPPV fatigue was seen in both groups A and B.

Using these MSPEVs, we calculated the ratio of the MSPEV of the positional nystagmus induced by the second Dix–Hallpike test against that induced by the first Dix–Hallpike test, and the ratio of the MSPEV of the positional nystagmus induced by the reverse Dix–Hallpike test against that induced by the first Dix–Hallpike test, in groups A and B (Figure 4B). The ratio of the MSPEV of the positional nystagmus induced by the second Dix–Hallpike test against that induced by the first Dix–Hallpike test in group A was significantly higher than that in group B (*p* = 0.0015, Mann–Whitney *U*-test). Thus, the effect of BPPV fatigue was weaker in group A compared with group B. There were no differences in the ratio of the MSPEV of the positional nystagmus induced by the reverse Dix–Hallpike test against that induced by the first Dix–Hallpike test between group A and B (*p* = 0.2268, Mann–Whitney *U*-test). To calculate the correlation coefficient between the MSPEV of the positional nystagmus induced by the first Dix–Hallpike test and that induced by the reverse Dix–Hallpike test, we used a graph obtained by plotting the MSPEV of the positional nystagmus induced by the first Dix–Hallpike test on the horizontal axis and that induced by the reverse Dix–Hallpike test on the vertical axis (Figure 4C). The MSPEV of the positional nystagmus induced by the reverse Dix–Hallpike test increased proportionally to the increase induced by the first Dix–Hallpike test. The correlation coefficient (R) was 0.5586. Overall, these data (Figures 4B,C) suggest that the amplitude of the MSPEV of the positional nystagmus induced by the reverse Dix–Hallpike test was dependent on the amplitude of that induced by the first Dix–Hallpike test, regardless of the presence/absence of the 10-min interval time in the supine position with the head turned to the affected side.

## Discussion

We previously reported that the effect of BPPV fatigue disappears within 30 min when maintaining a sitting head position (i.e., the intensity of the positional nystagmus weakened by BPPV fatigue recovers to the original intensity after 30 min in the sitting head position) (3). Thus, when the head is maintained in the sitting position, the effect of BPPV fatigue gradually reduces over 30 min. In the present study, we examined whether the effect of BPPV fatigue is weakened by other head positions (e.g., the supine position with the head turned to the affected side) during the Dix–Hallpike test. We compared the ratio of the MSPEV of the positional nystagmus induced by the second Dix–Hallpike test against that induced by the first Dix–Hallpike test between group A and B. As shown in Figure 1, a 10-min interval time was set for the sitting head position after the reverse Dix–Hallpike test in group A, while a 10-min interval time was set for the supine position with the head turned to the affected side after the first Dix–Hallpike test in group B. The ratio in group B was statistically smaller than that for group A (left graph of Figure 4B), and the ratio was very small (average value, 0.07). Thus, the effect of BPPV fatigue was maintained in the supine position with the head turned to the affected side. Overall, these data suggest that the effect of BPPV fatigue is maintained in the supine position with the head turned to the affected side, while the effect is reduced in the sitting head position.

**Figure 5:**
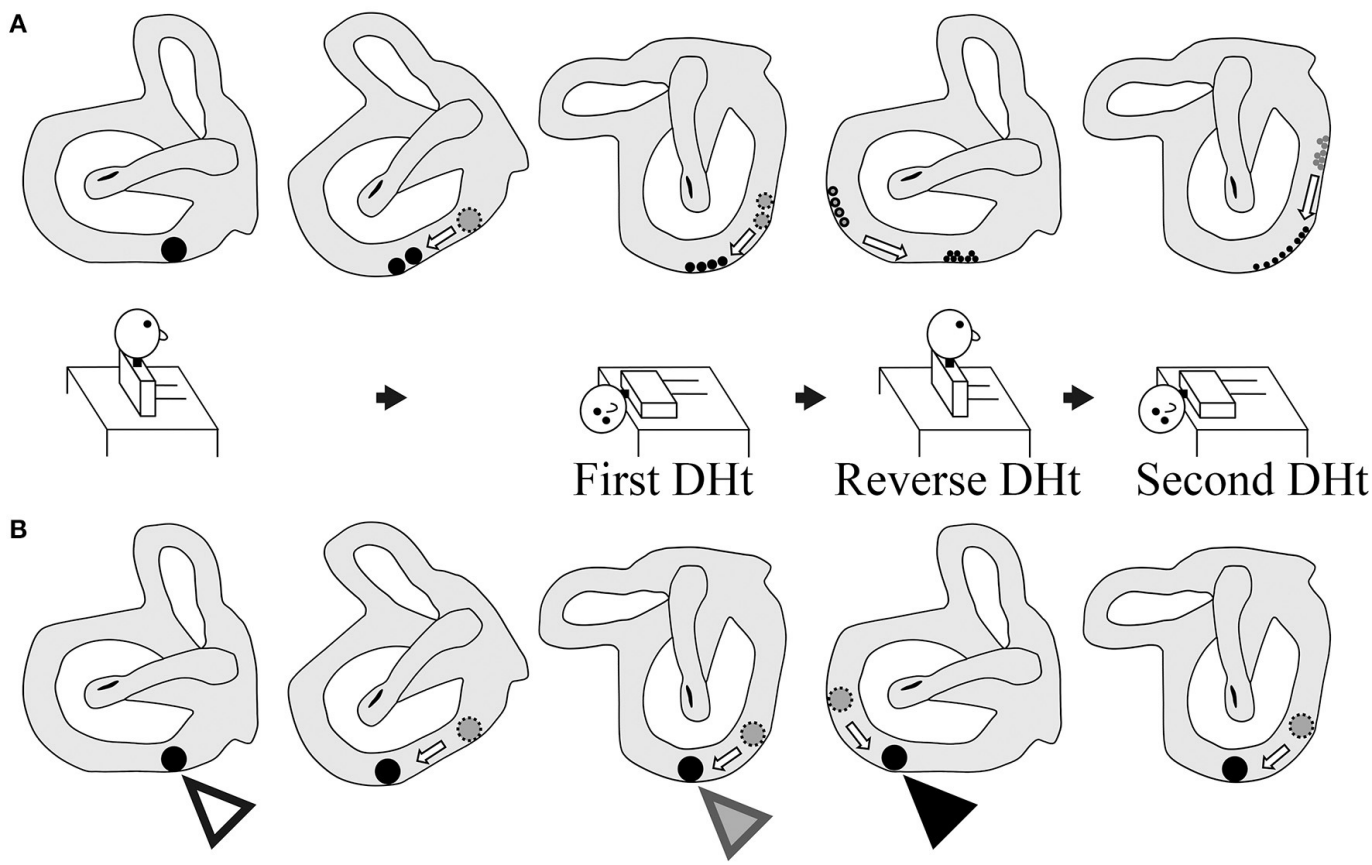
Two theories of the pathophysiology of BPPV fatigue. **(A)** Popular theory. As the size of the otoconial debris before the second Dix–Hallpike test is smaller than that before the first Dix–Hallpike test, the positional nystagmus is weaker following the second Dix–Hallpike test. Thus, the pathophysiology of BPPV fatigue involves dispersal of the otoconial debris in a semicircular canal during the head movement in the Dix–Hallpike test. **(B)** Boselli theory. The initial position of the otoconial debris before the first Dix–Hallpike test is shown by a white triangle. The position of the otoconial debris after the head movement of the first Dix–Hallpike test is shown by a gray triangle. From this head position, the patient's head is moved to the sitting position by the reverse Dix–Hallpike test, and the position of the otoconial debris when in the sitting position (i.e., just before performing the second Dix–Hallpike test) is shown by a black triangle. The second trajectory of the otoconial debris following the second Dix–Hallpike test is shown in the right figure. The trajectory of the otoconial debris is shorter than that for the first trajectory at the first Dix–Hallpike test (the first trajectory is between the white and gray triangles). The movement of the otoconial debris along the second short trajectory causes smaller cupula displacements than that for the first movement along the first long trajectory. Thus, the intensity of the positional nystagmus induced by the second Dix–Hallpike test is smaller than that following the first Dix–Hallpike test. As such, the pathophysiology of BPPV fatigue involves the otoconial debris not returning to its initial position after the first Dix–Hallpike test.

**Figure 6:**
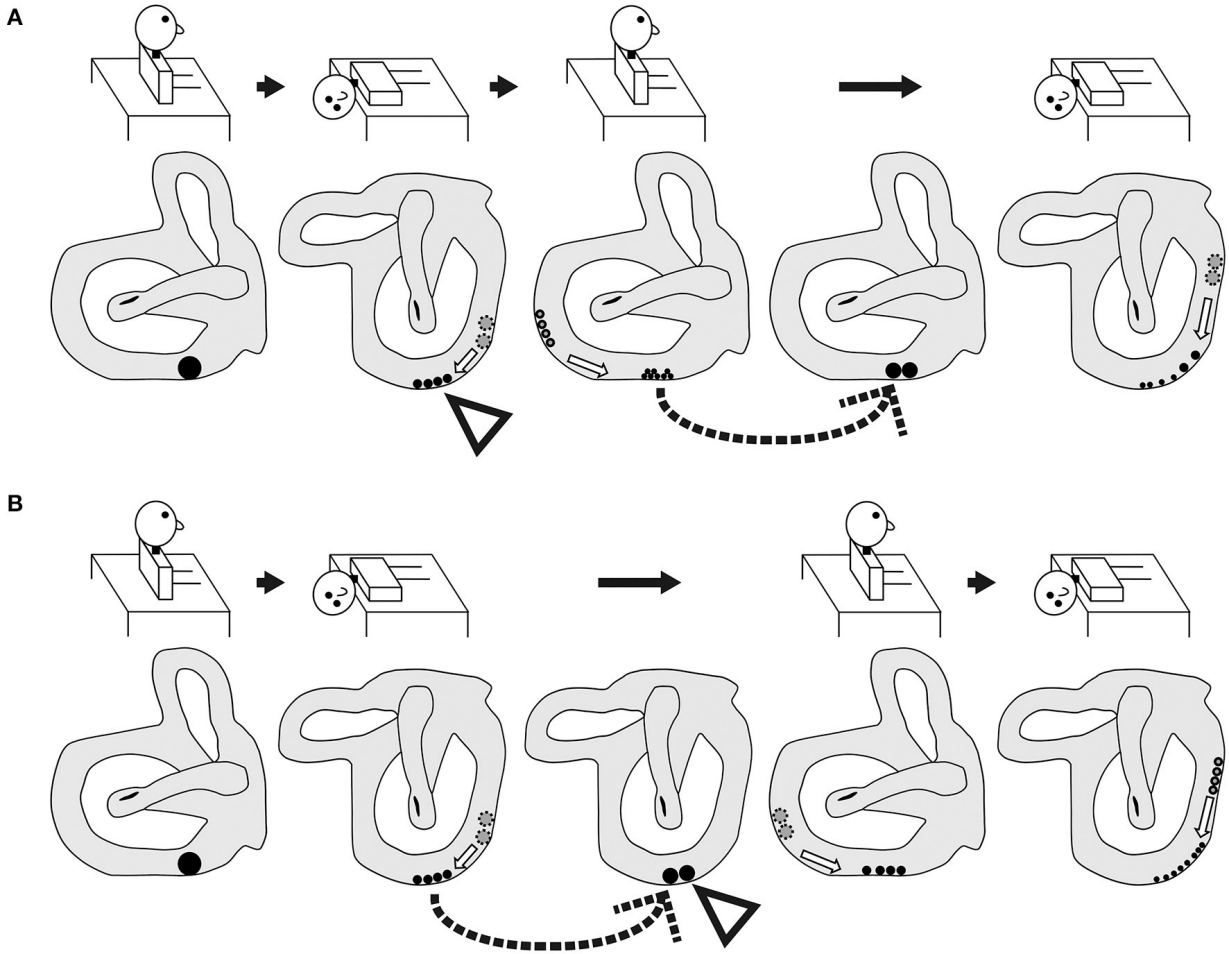
Schematic of the expected shape and movement of otoconial debris based on the popular theory of the pathophysiology of BPPV fatigue. **(A)** Group A. As shown by the dotted arrow, the dispersed otoconial debris returns to a mass during the sitting head position following a 10-min interval after the reverse Dix–Hallpike test. Therefore, strong positional nystagmus should be induced by the second Dix–Hallpike test as the mass of the otoconial debris (rather than dispersed otoconial debris) moves along the semicircular canal during head movement in the second Dix–Hallpike test. **(B)** Group B. As shown by the dotted arrow, the dispersed otoconial debris returns to a mass during the supine position with the head turned to the affected side following a 10-min interval after the first Dix–Hallpike test. Therefore, strong positional nystagmus should be induced by the reverse Dix–Hallpike test as the mass of otoconial debris (rather than dispersed otoconial debris) moves along the semicircular canal during the head movement in the reverse Dix–Hallpike test.

Based on these findings, we examined the pathophysiology of BPPV fatigue. According to the most well-established theory (16), as the otoconial debris become dispersed along the canal during the head movement of the first Dix–Hallpike test, they become less effective in creating endolymph drag and cupular deflection after the second Dix–Hallpike test (Figure 5A). This theory is based on physio-mathematical models in which otoconia within the affected semicircular canal reaching a “critical mass” is a prerequisite for BPPV (1, 17, 18). In the present study, the dispersed otoconial debris returned to a mass after maintaining the sitting position for 10 min (dotted arrow in Figure 6A). Thus, when the second Dix–Hallpike test was performed after a delay from the first Dix–Hallpike test, the intensity of the positional nystagmus induced by the second Dix–Hallpike test returned to that induced by the first Dix–Hallpike test (Figure 2A vs. 2C, Figure 3B vs. 3D, left graph in Figure 4A) (3).

However, our finding in the right graph of Figure 4B could not be explained by this theory. For example, according to this theory, in group B, the dispersed otoconial debris should return to a mass during the 10-min interval in the supine position with the head turned to the affected side (dotted arrow of Figure 6B), in the same way that in group A, the dispersed otoconial debris return to a mass during 10-min interval in the sitting head position (dotted arrow of Figure 6A). Thus, the size of the otoconial debris just before performing the reverse Dix–Hallpike test in group B should be larger than that in group A, and the MSPEV of the positional nystagmus induced by the reverse Dix–Hallpike test in group B should be larger than that in group A (white triangles in Figures 6A,B). As such, the ratio of the MSPEV of the positional nystagmus induced by the reverse Dix–Hallpike test against that induced by the first Dix–Hallpike test in group B should be larger than that in group A. However, we found that the value of the MSPEV of the positional nystagmus induced by the reverse Dix–Hallpike test depended only on the value of the MSPEV of the positional nystagmus induced by the first Dix–Hallpike test, not on the allocated group (right graph of Figures 4B,C). Thus, we suggest that otoconial debris is not dispersed or that the dispersed otoconial debris does not return to a mass.

Boselli et al. (2) proposed an alternative theory to explain the pathophysiology of BPPV fatigue. In this theory, the otoconial debris do not return to their initial position after the first Dix–Hallpike test. As a result, the second Dix–Hallpike test causes movement of the otoconial debris with a different trajectory. This causes smaller cupula displacements, even though the patients make the same head movements in the first and second Dix–Hallpike tests (Figure 5B). This theory can explain our findings, as follows. When the sitting head position is maintained for a certain time after the first Dix–Hallpike test (e.g., patients in group A), the otoconial debris move from the position shown by black triangle in Figure 5B to that shown by the white triangle in Figure 5B. As such, following the second Dix–Hallpike test, the trajectory of the otoconial debris in group A is longer than that in group B, and the MSPEV of the positional nystagmus induced by the second Dix–Hallpike test in group A is larger than that in group B. Thus, the ratio of the MSPEV of the positional nystagmus induced by the second Dix–Hallpike test against that following the first Dix–Hallpike test in group A was larger than that in group B (left graph of Figure 4B). Both with or without the 10-min interval in the supine position with the head turned to the affected side, the otoconial debris just prior to performing the reverse Dix–Hallpike test is shown by the gray triangle in Figure 5B. As a result, regardless of whether there is an interval (i.e., group A vs. B), the trajectory of the otoconial debris following the reverse Dix–Hallpike test is similar, resulting in a similar MSPEV of the positional nystagmus induced by the reverse Dix–Hallpike test in groups A and B. Indeed, we found no differences between the ratio of the MSPEV of the positional nystagmus induced by the reverse Dix–Hallpike test against that induced by the first Dix–Hallpike test between group A and B (right graph in Figure 4B).

Overall, our data support the theory of the pathophysiology of BPPV fatigue proposed by Boselli et al. Nevertheless, as the MSPEV of the positional nystagmus induced by the second Dix–Hallpike test in group B was very small (right graph in Figure 4A), this theory would predict that the length between the white and black triangles in Figure 5B is long. Therefore, the shape of the posterior canal must be distorted, rather than in a smooth circle. However, in reality, the shape of the posterior canal is almost a smooth circle (19). Therefore, the theory of Boselli et al. is not able to fully explain the pathophysiology of BPPV fatigue. Further, studies are required to clarify the exact pathophysiology of BPPV fatigue.

We recommend that when checking for the presence of BPPV fatigue, a second Dix–Hallpike test should be performed soon after the first Dix–Hallpike test. Furthermore, to clearly observe positional nystagmus, the second Dix–Hallpike test should be performed more than 10 min after the first Dix–Hallpike test (during the 10 min between the tests, the patients should maintain a sitting position and not a supine position).

The limitations of this study are as follows. First, the study sample size was small. Second, although, we discussed the possibility that the shape of the posterior canal was distorted, we did not perform imaging to assess the shape of the posterior canal. Without imaging data, it is difficult to predict the anatomical characteristics or variations of the posterior semicircular canal in each participant.

## Conclusion

The effect of BPPV fatigue is continued by maintaining a supine position with the head turned to the affected side, while the effect is weakened by maintaining a sitting head position. On the basis of the most widely accepted theory of the pathophysiology of BPPV fatigue, in which the particles become dispersed along the canal during head movement in the Dix–Hallpike test, we found an inconsistency whereby the dispersed otoconial debris return to a mass during the sitting position but do not return to a mass in the supine position with the head turned to the affected side. Future studies are required to determine the exact pathophysiology of BPPV fatigue.

## Data Availability Statement

The original contributions presented in the study are included in the article/supplementary material, further inquiries can be directed to the corresponding author/s.

## Ethics Statement

The studies involving human participants were reviewed and approved by Osaka University Hospital. The patients/participants provided their written informed consent to participate in this study.

## Author Contributions

TI substantially contributed to conception of the study and drafting of the article. TI, SN, TS, YO, and TK substantially contributed to acquisition of data. TI and TO substantially contributed to analysis of data. TI and NT substantially contributed to interpretation of data. HI substantially contributed to study supervision. All authors contributed to the article and approved the submitted version.

## Conflict of Interest

The authors declare that the research was conducted in the absence of any commercial or financial relationships that could be construed as a potential conflict of interest.
